# Heterogeneity and Adjuvant Therapeutic Approaches in MSI-H/dMMR Resectable Gastric Cancer: Emerging Trends in Immunotherapy

**DOI:** 10.1245/s10434-023-14103-0

**Published:** 2023-09-04

**Authors:** Hui Wu, Wenyuan Ma, Congfa Jiang, Ning Li, Xin Xu, Yongfeng Ding, Haiping Jiang

**Affiliations:** 1 Department of Medical Oncology, The First Affiliated Hospital, Zhejiang University School of Medicine, Hangzhou, 310006 China; 2 Zhejiang University School of Medicine, Hangzhou, China; 3 Department of Hematology and Oncology, Ningbo Forth Hospital, Ningbo, China

**Keywords:** Gastric cancer, Microsatellite instability-high/deficient mismatch repair, Heterogeneity, Adjuvant chemotherapy, Immunotherapy

## Abstract

Gastric cancer (GC) remains one of the world’s most common and fatal malignant tumors. With a refined understanding of molecular typing in recent years, microsatellite instability (MSI) has become a major molecular typing approach for gastric cancer. MSI is well recognized for its important role during the immunotherapy of advanced GC. However, its value remains unclear in resectable gastric cancer. The reported incidence of microsatellite instability-high (MSI-H)/deficient mismatch repair (dMMR) in resectable gastric cancer varies widely, with no consensus reached on the value of postoperative adjuvant therapy in patients with MSI-H/dMMR resectable GC. It has been established that MSI-H/dMMR tumor cells can elicit an endogenous immune antitumor response and ubiquitously express immune checkpoint ligands such as PD-1 or PD-L1. On the basis of these considerations, MSI-H/dMMR resectable GCs are responsive to adjuvant immunotherapy, although limited research has hitherto been conducted. In this review, we comprehensively describe the differences in geographic distribution and pathological stages in patients with MSI-H/dMMR with resectable gastric cancer and explore the value of adjuvant chemotherapy and immunotherapy on MSI-H/dMMR to provide a foothold for the individualized treatment of this patient population.

**Supplementary Information:**

The online version contains supplementary material available at 10.1245/s10434-023-14103-0.

Gastric cancer (GC) is an aggressive and heterogeneous malignancy with limited therapeutic options available, especially in locally advanced and metastatic stages, accounting for the poor prognosis of affected patients.^1^ Less than 50% of patients are diagnosed with early stage disease, and surgical resection plus lymphadenectomy remains the mainstay of curative treatment. Surgical resection with subsequent adjuvant chemotherapy has been established as the standard-of-care treatment for patients with stages II and III gastric cancer, which is more beneficial than surgery alone in terms of overall survival (OS).^2^ Unfortunately, approximately 40–60% of patients who undergo resection relapse and die from cancer.^3–5^

With significant progress achieved in molecular research on gastric cancer, many researchers have attempted to explore the underlying mechanisms and inherent characteristics of the genome, transcriptome, and protein expression of gastric carcinomas, and have successfully proposed several molecular subtyping systems.^5–7^ According to the Cancer Genome Atlas (TCGA) and Asian Cancer Research Group (ACRG), the microsatellite instability (MSI) subtype represents an important molecular subtype of gastric cancer. Microsatellites (MS) are DNA regions prone to mutations, usually assessed by polymerase chain reaction (PCR)/next-generation sequencing (NGS).^8^ An increasing body of evidence suggests that the mismatch repair (MMR) system measured by immunohistochemistry (IHC) is responsible for monitoring and correcting errors in DNA replication.^9–11^ Studies by Kim et al.^12^ and Smyth et al.^13^ evaluated the consistency of microsatellite status and MMR protein expression in gastric cancer and concluded that microsatellite instability-high (MSI-H) was generally strongly correlated with deficient mismatch repair (dMMR).

The proportion of MSI-H/dMMR in resectable gastric cancer exhibits significant heterogeneity across different studies. The prevalence of MSI-H in patients with gastric cancer was 23.5% (*n* = 111/472) in Italy^14^ but only 8.5% (*n* = 170/1990) in South Korea.^15^ In recent years, the value of adjuvant therapy in MSI-H/dMMR colon cancers has become increasingly well established. In this respect, the National Comprehensive Cancer Network (NCCN) guidelines for colon cancer^16^ classify patients with MSI-H/dMMR as low risk and have recommended that patients with stage II disease and MSI-H do not require adjuvant therapy since 2010. In the context of MSI-H/dMMR gastric cancer, the existing data in the adjuvant setting are still pauce, and the existing guidelines from leading organizations such as NCCN, CSCO, and ESMO do not offer definitive recommendations regarding the appropriate selection of adjuvant treatment. Although the potential of adjuvant therapy in MSI-H/dMMR resectable gastric cancer has been poorly explored, the effectiveness of immunotherapy has been demonstrated in MSI-H/dMMR advanced gastric cancer. Immune checkpoint inhibitors (ICIs) have been integrated into the NCCN gastric cancer guidelines as a second-line or subsequent treatment for MSI-H/dMMR advanced gastric cancer and have even been suggested as the first-line therapy by the Chinese Society of Clinical Oncology (CSCO) guidelines,^17^ although their value in the setting of MSI-H/dMMR resectable gastric cancer has not been confirmed. Several small-scale clinical trials are currently underway to predict the value of immunotherapy in MSI-H/dMMR resectable gastric cancer, but no results have been reported yet.

In this review, we focus on the geographical variations and differences in pathological stages of MSI-H/dMMR resectable gastric cancer on the basis of the latest literature, and highlight the potential of adjuvant chemotherapy and immunotherapy for patients with MSI-H/dMMR resectable GC.

## MSI-H/dMMR GCs: Geographical Differences

**Fig. 1:**
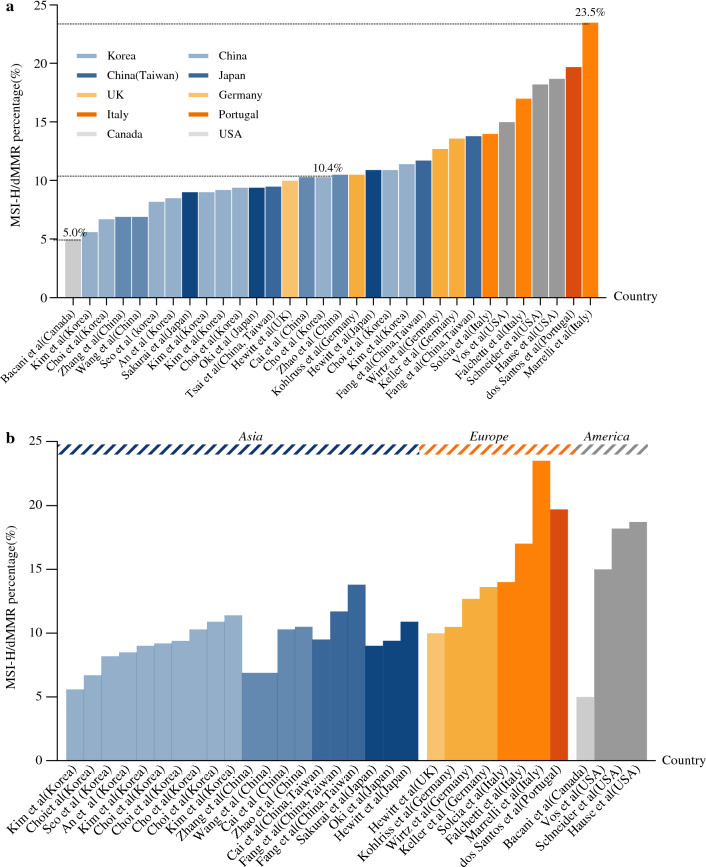
Heterogeneity of MSI-H/dMMR in geographical distribution. **a** Incidence of MSI-H/dMMR among countries. **b** Heterogeneity of MSI-H/dMMR in geographical distribution among Asian, European, and American countries

**Fig. 2:**
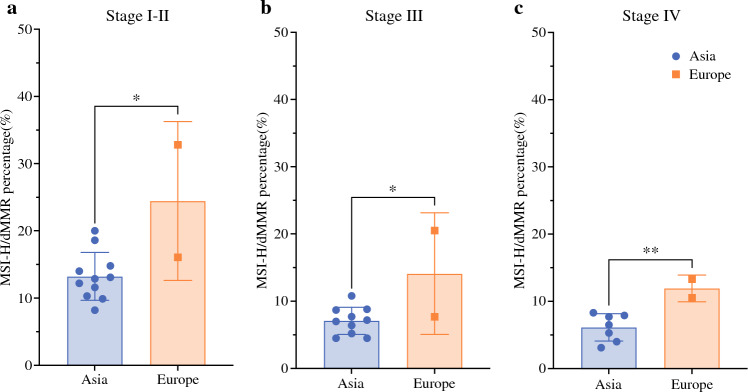
Regional differences of MSI-H/dMMR proportion in each stage. **a** Proportion of MSI-H/dMMR in Asia vs. Europe in stages I–II (*P = 0.0128, unpaired *t*-test). **b** Proportion of MSI-H/dMMR in Asia vs. Europe in stage III (*P = 0.0253, unpaired *t*-test). **c** Proportion of MSI-H/dMMR in Asia vs. Europe in stage IV (**P = 0.0092, unpaired *t-*test)

Gastric cancer (GC) accounts for the second highest mortality among cancers worldwide, with higher prevalence in East Asia, especially in Japan, China, South Korea, and some developing countries.^7,18^ To identify relevant studies, a comprehensive literature search was conducted in PubMed and Web of Science databases (Supplementary Methods, Fig. S1). Given the high concordance with MSI-H, dMMR was also included at retrieval. To enhance the accuracy of our review, we restricted our analysis to studies with a sample size greater than 50. As a result, we identified 31 records that reported the frequencies of MSI-H or dMMR in gastric cancer. Our retrospective analysis of studies conducted over the past few years indicated that the incidence of MSI-H/dMMR varied among countries, ranging from 5.0 to 23.5%.^9,14,15,19–46^ (Table 1 and Fig. 1a). It has been established that the proportion of MSI-H/dMMR gastric cancer in Asia is lower than in European countries (Fig. 1b). In this respect, the median proportion of MSI-H/dMMR GC is 9.3% and 13.8% in Asian and European countries, respectively. Meanwhile, the proportion of MSI-H/dMMR in different nations was similar but differed considerably in America (Fig. S2). A study from Canada reported that the prevalence of MSI-H/dMMR was 5% (*n* = 7/139),^20^ while in other research from North America, MSI-H/dMMR was found in 18.7% (*n* = 52/278) of patients.^45^ This disparity may be attributed to the relatively small number of patients enrolled in each study. Moreover, considering that the frequency of MSI-H/dMMR may vary by stage, which was scientifically valid in the case of colorectal cancer,^47,48^ we adjusted for any such stage differences when examining regional differences. The proportion difference in each stage between Asian and European populations is plotted in Fig. 2a–c, and stage I and stage II were combined as the early stage of gastric cancer because of the small amount of literature. Interestingly, the proportion of MSI-H/dMMR in Europe was significantly higher than in Asia, irrespective of the cancer stage (*P* < 0.05).

**Table 1:** Heterogeneity of MSI-H/dMMR in geographical distribution and pathological stage

	References	Country	No. of patients	Method	MSS/MSI-L	MSI-H/dMMR	Proportion of MSI-H/dMMR in each pTNM stage				
							Stage I	Stage II	Stage III	Stage IV	
Asia	An et al. 15	Korea	1990	PCR	1820 (91.5%)	170 (8.5%)	109/1194 (9.1%)	37/288 (12.8%)	17/377 (4.5%)	7/131 (5.3%)	
	Kim et al. 21	Korea	414	PCR	391 (94.4%)	23 (5.6%)					
	Kim et al. 22	Korea	1786	PCR	1625 (91.0%)	161 (9.0%)					
	Seo et al. 23	Korea	328	PCR	301 (91.8%)	27 (8.2%)	13/183 (7.1%)	6/48 (12.5%)	7/65 (10.8%)	1/32 (3.1%)	
	Choi et al. ^24^	Korea	592	PCR	552 (93.2%)	40 (6.7%)		24/282 (8.5%)	16/310 (5.2%)		
	Kim et al. 25	Korea	359	PCR	318 (88.6%)	41 (11.4%)	11/76 (14.5%)	18/145 (12.4%)	12/138 (8.7%)		
	Choi et al. ^26^	Korea	459	PCR	416 (90.6%)	43 (9.4%)	22/293 (7.5%)	16/75 (21.3%)	3/67 (4.5%)	2/24 (8.3%)	
	Choi et al. 27	Korea	623	PCR	555 (89.1%)	68 (10.9%)	44/384 (11.5%)	13/80 (16.3%)	9/128 (7.0%)	2/31 (6.5%)	
	Kim et al. 28	Korea	1178	IHC	1070 (90.8%)	108 (9.2%)					
	Cho et al. 29	Korea	580	IHC	520 (89.7%)	60 (10.3%)	1/11 (9.1%)	25/119 (21.0%)	17/235 (7.2%)	17/215 (7.9%)	
	Zhao et al. 30	China	210	PCR	188 (89.5%)	22 (10.5%)	8/31 (25.8%)	5/62 (8.1%)	8/104 (7.7%)	1/13 (7.7%)	
	Cai et al. 31	China	271	PCR IHC	243 (89.7%)	28 (10.3%)	19/102 (18.6%)		9/169 (5.3%)		
	Zhang et al. 32	China	2031	NGS IHC	1891 (93.1%)	140 (6.9%)					
	Wang et al. 33	China	2472	PCR IHC	2301 (93.1%)	171 (6.9%)					
	Fang et al. 34	China (Taiwan)	214	PCR	189 (88.3%)	25 (11.7%)	6/68 (8.8%)	12/71 (16.9%)	9/140 (6.4%)		
	Fang et al. 35	China (Taiwan)	326	PCR	281 (86.2%)	45 (13.8%)					
	Tsai et al. 36	China (Taiwan)	1206	IHC	1092 (90.5%)	114 (9.5%)	31/277 (11.2%)	25/206 (12.1%)	53/599 (8.8%)	5/124 (4.0%)	
	Oki et al. 19	Japan	240	PCR	218 (90.6%)	22 (9.4%)	12/98 (12.2%)		10/117 (8.5%)		
	Sakuraiet al. 37	Japan	167	PCR	152 (91.0%)	15 (9.0%)					
	Hewitt et al. 38	Japan	396	PCR IHC	353 (89.1%)	43 (10.9%)					
Europe	Marrelli et al. 14	Italy	472	PCR	361 (76.5%)	111 (23.5%)	18/75 (24.0%)	43/111 (38.7%)	41/200 (20.5%)	9/86 (10.5%)	
	Solcia et al. 39	Italy	294	PCR	253 (86.0%)	41 (14.0%)					
	Falchetti et al. 9	Italy	159	PCR	132 (83.0%)	27 (17.0%)					
	Wirtz et al. 40	Germany	126	PCR	110 (87.3%)	16 (12.7%)					
	Keller et al. ^41^	Germany	59	PCR	51 (86.4%)	8 (13.6%)	2/21 (9.5%)	3/10 (30.0%)	1/13 (7.7%)	2/15 (13.3%)	
	Kohlruss et al. 42	Germany	143	PCR	128 (89.5%)	15 (10.5%)					
	dos Santos et al. 43	Portugal	61	PCR	49 (80.3%)	12 (19.7%)					
	Hewitt et al. 38	UK	702	PCR IHC	632 (90.0%)	70 (10.0%)					
America	Bacani et al. 20	Canada	139	PCR	132 (95.0%)	7 (5.0%)					
	Schneider et al. 44	USA	143	PCR	117 (81.8%)	26 (18.2%)					
	Hause et al. 45	USA	278	PCR	226 (81.3%)	52 (18.7%)					
	Vos et al. 46	USA	535	NGS IHC	453 (85.0%)	82 (15.0%)					
The sample size of the studies is more than 50.											

*MSS* microsatellite stability, *MSI-L* microsatellite instability-low, *MSI-H* microsatellite instability-high, *dMMR* deficient mismatch repair,

*PCR* polymerase chain reaction, *IHC* immunohistochemistry, *NGS* next-generation sequencing

## MSI-H/dMMR GCs: Differences in Pathological Stage

**Fig. 3:**
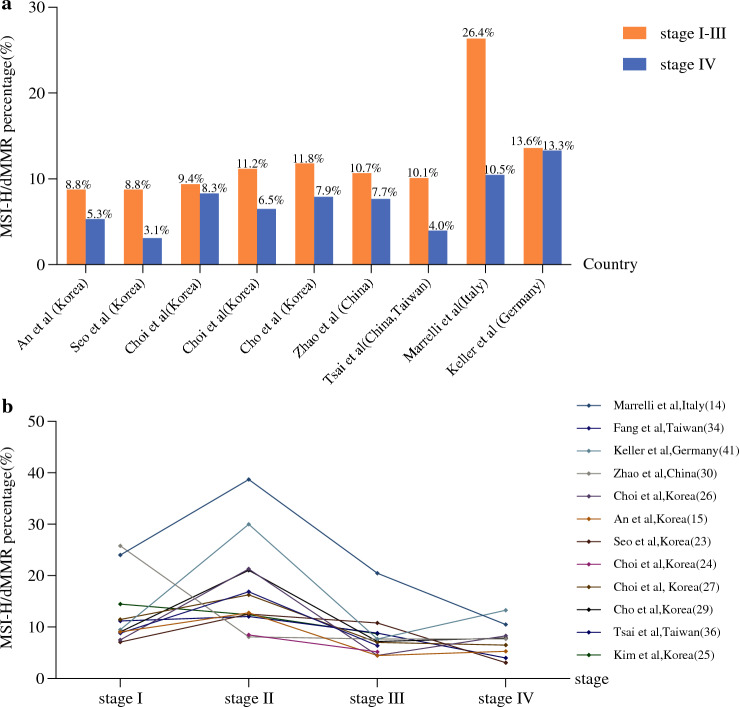
Heterogeneity of MSI-H/dMMR in pathological stages. **a** Prevalence of MSI-H/dMMR between resectable and advanced GCs. **b** Prevalence of MSI-H/dMMR among four pathological stages. *MSI-H* microsatellite instability-high, *dMMR* deficient mismatch repair, *GCs* gastric cancers

Our literature review showed that 14 of 31 studies further staged patients with MSI-H/dMMR according to the American Joint Committee on Cancer (AJCC) staging system (Table 1). Although MSI-H/dMMR can be observed in all gastric cancer stages, the proportion of MSI-H/dMMR in resectable gastric cancer was generally higher than in stage IV disease (Table 2, Fig. 3a). Interestingly, in approximately 80% of included studies, the prevalence of MSI-H/dMMR in stage II was the highest (Fig. 3b). For instance, in a large-scale study published in 2012, tissue specimens from 1990 patients with gastric cancer were collected and analyzed using PCR amplification.^15^ The prevalence of MSI-H/dMMR was the highest in stage II GC (12.8%), followed by stages I (9.1%), IV (5.3%), and III (4.5%). An increasing body of evidence from recently published studies^29,36^ suggests that the incidence of MSI-H/dMMR disease in patients with gastric cancer is higher in stage II than in other stages. Although the proportion of MSI-H/dMMR in resectable gastric cancers was higher than in stage IV, the effect of postoperative adjuvant therapy in resectable gastric cancer remains unclear, warranting further exploration.

**Table 2:** Prevalence of MSI-H/dMMR between resectable and advanced GCs

References	Country	No. of patients	MSS/MSI-L	MSI-H/dMMR	Proportion of MSI-H/dMMR in stages I–III vs. stage IV	
					Stages I–III	Stage IV
An et al. 15	Korea	1990	1820 (91.5%)	170 (8.5%)	163/18 59(8.8%)	7/131 (5.3%)
Seo et al. 23	Korea	328	301(91.8%)	27 (8.2%)	26/296 (8.8%)	1/32 (3.1%)
Choi et al. 26	Korea	459	416 (90.6%)	43 (9.4%)	22/435 (9.4%)	2/24 (8.3%)
Choi et al. 27	Korea	623	555 (89.1%)	68 (10.9%)	66/592 (11.2%)	2/31 (6.5%)
Cho et al. 29	Korea	580	520(89.7%)	60 (10.3%)	43/365 (11.8%)	17/215(7.9%)
Zhao et al. 30	China	210	188(89.5%)	22 (10.5%)	21/197 (10.7%)	1/13(7.7%)
Tsai et al. 36	China (Taiwan)	1206	1092 (90.5%)	114 (9.5%)	109/1082 (10.1%)	5/124 (4.0%)
Marrelli et al. 14	Italy	472	361 (76.5%)	111 (23.5%)	102/386 (26.4%)	9/86 (10.5%)
Keller et al. 41	Germany	59	51 (86.4%)	8 (13.6%)	6/44 (13.6%)	2/15 (13.3%)

*MSS* microsatellite stability, *MSI-L* microsatellite instability-low, *MSI-H* microsatellite instability-high, *dMMR* deficient mismatch repair

## Adjuvant Chemotherapy of MSI-H/dMMR Resectable GCs: Opportunity or Hindrance?

With adjuvant chemotherapy based on fluorouracil being guideline-endorsed for stage II/III resectable GCs, researchers have been increasingly interested in the sensitivity of chemotherapy drugs to MSI-H/dMMR gastric cancer (Table 3). The MAGIC trial^13^ conducted in 2017 (ECF neoadjuvant scheme: epirubicin + cisplatin + fluorouracil) and the CLASSIC trial^24^ in 2019 (XELOX adjuvant scheme: capecitabine plus oxaliplatin) both concluded that patients with MSI-H/dMMR gastric cancer do not benefit from perioperative or postoperative chemotherapy. In addition, a meta-analysis involving samples from four large randomized clinical trials (MAGIC, CLASSIC, ARTIST, and ITACA-S) revealed no significant benefit in OS at 5 years (75.4% in the postoperative chemotherapy arm vs. 82.8% in the only-surgery arm).^49^ The previously mentioned studies advocated that MSI-high gastric cancers were less likely to respond to chemotherapy.

**Table 3:** The adjuvant chemotherapy effect of MSI-H/dMMR in GC in different treatment settings

References	Country	Study design	Tumor type	No. of patients		Drugs	5-year survival of OS/DFS	Hazard rate (HR)	Results
				MSI-H/dMMR percentage	Chemo-therapy vs. surgery alone				
Choi et al. 24	Korea	Post hoc analysis of CLASSIC RCT	Stage II/III resectable gastric cancer	40 (6.8%) using PCR	19 vs. 21	Capecitabine plus oxaliplatin(XELOX)	DFS 84.8% vs. 85.7%	DFS 1.05 (0.21–5.18)	Adjuvant chemotherapy cannot benefit MSI-H
Tsai et al. 36	China (Taiwan)	Retrospective cohort analysis	Stage II-IV gastric cancer	83 (8.9%) using IHC	59 vs. 24	5-FU, titanium silicate-1, uracil-tegafur, or oxaliplatin plus capecitabine	DFS 61.2% vs. 55.9% OS 61.7% vs. 56.4%	DFS 0.64 (0.21–1.92) OS 0.80 (0.35–1.82)	Adjuvant chemotherapy cannot benefit MSI-H
Kim et al. 25	Korea	Retrospective cohort analysis	Stage II/III resectable gastric cancer	41 (11.4%) using PCR	82 vs. 75	Fluoropyrimidine plus platinum	DFS 81.1% vs. 73.7%	DFS 0.53 (0.27–1.04)	Adjuvant chemotherapy cannot benefit MSI-H
							OS 93.9% vs. 78.4%	OS 0.52 (0.26–1.03)	
Wang et al. 50	China	Retrospective cohort analysis	Stage II/III resectable gastric cancer	176 (26.3%) using IHC	79 vs. 97	Fluorouracil- based	OS 65.6% vs. 48.1%	OS 0.59 (0.38–0.93)	Adjuvant chemotherapy plus surgery has better survival
Yang et al. 51	China	Retrospective cohort analysis	Atage I/II/III gastric cancer	54 (23.9%) using IHC	101 vs. 125	Platinum/fluorouracil-based	OS 61.0% vs. 55.8%	OS 0.76 (0.34–1.70)	Adjuvant chemotherapy cannot benefit MSI-H

*MSI-H* microsatellite instability-high, *dMMR* deficient mismatch repair, *5-FU* 5-fluorouracil, *OS* overall survival, *DFS* disease-free survival, *RCT* randomized controlled trial

In recent years, the role of MSI status in predicting the chemotherapy response has been extensively studied. A retrospective study on patients with stage II/III MSI-H/dMMR GC published in 2020 predicted the efficacy of adjuvant therapy by creating a new immune scoring system (ISSGC), suggesting that patients with MSI-H/dMMR GC have longer OS with adjuvant chemotherapy after surgery [hazard ratio, HR 0.59 (0.38–0.93)].^50^ Notwithstanding that many other studies demonstrated a favorable trend with the implementation of adjuvant chemotherapy on patients with MSI-H/dMMR, the final results remained non-significant.^51^ For example, in a study performed by Tsai and colleagues,^36^ although patients who received adjuvant chemotherapy showed better 5 year disease-free survival (DFS) (61.2% vs. 55.9%), no significant benefit was found for postoperative adjuvant chemotherapy of MSI-H/dMMR gastric cancer [DFS, HR 0.64 (0.21–1.94) and OS, HR 0.8 (0.35–1.82)]. In a recent meta-analysis,^52^ seven studies were included to explore the prognostic impact of adjuvant chemotherapy. By using Cox models or fixed/random effects models to pool HR, it was found that patients with MSI-H/dMMR could benefit from adjuvant chemotherapy, with estimated HRs of 0.56 (95% CI 0.36–0.87; *P* = 0.010) for DFS and 0.62 (95% CI 0.45–0.83; *P* = 0.002) for OS.

No consensus has been reached on the value of MSI-H/dMMR as a predictor of the efficacy of adjuvant chemotherapy, mainly due to the low number of patients with MSI-H/dMMR, the non-standardization of MSI detection standards, and the retrospective nature of most studies in the literature. In addition, only one prospective clinical trial (NCT03485196)^53^ analyzed the relationship between MSI status and efficacy of 5-fluorouracil (5-FU)-based adjuvant chemotherapy by observing the survival period of patients with different MSI statuses. This multicohort trial initiated by the Affiliated Hospital of Qingdao University in China involved 1000 patients with gastric cancer and was completed in August 2022. On the basis of the results, it was concluded that there was no significant difference in 1- and 2-year disease-free survival (DFS) rates after adjuvant chemotherapy among different MSI statuses, including MSI-H and dMMR, regardless of the specific adjuvant chemotherapy regimen used, such as XELOX, SOX, or other adjuvant treatment protocols.

## Immunotherapy of MSI-H/dMMR Resectable GCs: A Possible New Approach

The past few years have witnessed a burgeoning interest in immunotherapy, especially gastric cancer research (Table 4). On the basis of the results of the CheckMate 649^54^ and ORIENT-16^55^ trials, nivolumab or sintilimab combined with chemotherapy, as the first-line treatment of advanced gastric cancer, is more effective and safer than chemotherapy alone in China and abroad. Meanwhile, pembrolizumab or dostarlimab-gxly is recommended as a second-line or subsequent therapy for MSI-H/dMMR advanced gastric cancer by the 2022 NCCN guidelines.^2^

**Table 4:** Clinical trials on immune checkpoint inhibitors for MSI-H/dMMR advanced GC

Clinical trials	Status	Phase	Disease	Immune drug treatment	Result	Title
NCT01848834 (KEYNOTE-012)	Completed	I	PD-L1+ advanced gastric cancers	Pembrolizumab	MSI-H GC ORR 57.1% Overall GC ORR 22.0%	Study of Pembrolizumab (MK-3475) in Participants With Advanced Solid Tumors (MK-3475-012/KEYNOTE-012)
NCT02335411 (KEYNOTE-059)	Completed	II	Advanced gastric or GEJ cancers	Pembrolizumab	MSS GC ORR 9.0% MSI-H GC ORR 57.1% Overall GC ORR 11.6%	A Study of Pembrolizumab (MK-3475) in Participants With Recurrent or Metastatic Gastric or Gastroesophageal Junction Adenocarcinoma (MK-3475-059/KEYNOTE-059)
NCT02267343 (CheckMate-032)	Completed	III	Advanced gastric or GEJ cancers	Nivolumab	MSI-H GC ORR 29.0% MSS/MSI-L GC ORR 11.0%	Study of ONO-4538 in Unresectable Advanced or Recurrent Gastric Cancer NCT02370498
(KEYNOTE-061)	Completed	III	Advanced gastric or GEJ cancers	Pembrolizumab vs. paclitaxel	MSI-H GC median OS not reached vs. 8.1 months (HR 0.42; 95% CI 0.13–1.31)	A Study of Pembrolizumab (MK-3475) Vs. Paclitaxel for Participants With Advanced Gastric/Gastroesophageal Junction Adenocarcinoma That Progressed After Therapy With Platinum and Fluoropyrimidine (MK-3475-061/KEYNOTE-061)
NCT02494583 (KEYNOTE-062)	Completed	III	Advanced gastric or GEJ cancers	Pembrolizumab vs. Placebo+Cisplatin+5-Fluorouracil	MSI-H GC median OS not reached vs. 8.5 months Median PFS 11.2 vs. 6.6 months	Study of Pembrolizumab (MK-3475) as First-Line Monotherapy and Combination Therapy for Treatment of Advanced Gastric or Gastroesophageal Junction Adenocarcinoma (MK-3475-062/KEYNOTE-062)
NCT02628067 (KEYNOTE-158)	Recruiting	II	MSI-H gastric cancers	Pembrolizumab	MSI-H GC ORR:45.8% median PFS 11.0 months	Study of Pembrolizumab (MK-3475) in Participants With Advanced Solid Tumors (MK-3475-158/KEYNOTE-158)
NCT02915432	Active, not recruiting	Ib/II	Advanced gastric cancers	Toripalimab	TMB-H vs. TMB-L GC ORR 33.3% vs. 7.1%	The Study to Evaluate Toripalimab (JS001) in Patients With Advanced GC, ESCC, NPC, HNSCC
NCT05535569	Completed	Ib/II	Advanced gastric cancers	Nivolumab	N/A	Phase Ib/II Study to Evaluate the Safety and Efficacy of Nivolumab in Combination With Paclitaxel in Epstein-Barr Virus (EBV)-Related, or Microsatellite Instability-High (MSI-H), or Programmed Cell Death Ligand 1 (PD-L1) Positive Advanced Gastric Cancer
NCT03257163	Recruiting	II	dMMR gastric cancers	Pembrolizumab	N/A	Pembrolizumab, Capecitabine, and Radiation Therapy in Treating Patients With Mismatch-Repair Deficient and Epstein-Barr Virus Positive Gastric Cancer
NCT04817826	Recruiting	II	dMMR gastric cancers	DurvalumabTremelimumab	N/A	TremelImumab aNd Durvalumab For the Non-operatIve Management (NOM) of MSI-high Resectable GC/GEJC. (INFINITY)
NCT03236935	Active, not recruiting	I	Gastric cancers	L-NMMA Pembrolizumab	N/A	Phase Ib of L-NMMA and Pembrolizumab
NCT05572684	Recruiting	Ib/II	MSI-H gastric cancers	NC410 Pembrolizumab	N/A	A Safety, Tolerability and Efficacy Study of NC410 Plus Pembrolizumab in Participants With Advanced Unresectable or Metastatic Solid Tumors
NCT05177133	Recruiting	II	dMMR esophagogastric Cancer	Retifanlimab	N/A	Anti-PD-1 and CapOx for the First-line Treatment of dMMR Esophagogastric Cancer (AuspiCiOus)

*MSI-H* microsatellite instability-high, *dMMR* deficient mismatch repair, *GEJ cancers* gastroesophageal junction cancers, *PFS* progression-free survival, *ORR* overall response rate, *TMB-H* tumor mutation burden-high, *TMB-L* tumor mutation burden-low, *ESCC* esophageal squamous cell carcinoma, *NPC* nasopharyngeal carcinoma, *HNSCC* head and neck squamous cell carcinoma, *CapOx* capecitabine plus oxaliplati

Few clinical trials assessing adjuvant immunotherapy for resectable MSI-H/dMMR gastric cancer are currently underway. However, in the phase III IMpower010 study,^56^ anti-PD-L1 atezolizumab adjuvant treatment was found to improve the progression-free survival (PFS) (HR 0.79) and DFS (HR 0.66) of patients with resectable non-small cell lung cancer (NSCLC). As for postoperative adjuvant immunotherapy of resectable MSI-H/dMMR gastric cancer, we only identified two ongoing clinical trials (Table 5). The NCT05468138 study conducted by Fudan University in Shanghai aims to assess the efficacy of PD-1 antibody as an adjuvant treatment for patients with MSI-H/dMMR gastric cancer after D2 radical surgery. The PD-1 antibody (sintilimab or nivolumab) was set as the experimental group, and the standard chemotherapy regimen (SOX, XELOX) as the control group to compare the 3-year DFS of different adjuvant chemotherapy schemes. Another trial from Shanghai Tongji University (NCT04152889) is being carried out to evaluate the efficacy of camrelizumab in combination with docetaxel + S-1 sequenced by camrelizumab + S-1 in patients with stage III gastric cancer. Patients with stage III PD-L1+/MSI-H/EBV+/dMMR gastric cancer were included to explore the efficacy of combined immunochemotherapy. Although immunoadjuvant therapy can significantly prolong the survival of patients with NSCLC, further data on whether patients with MSI-H/dMMR resectable gastric cancer could benefit from immunoadjuvant treatment is required.

**Table 5:** Clinical trials on immune checkpoint inhibitors for MSI-H/dMMR resectable GC

Clinical trials	Status	Phase	Disease	Immune drug	Treatment	Title
Clinical trials on immune checkpoints inhibitors for MSI-H/dMMR resectable GC in adjuvant chemotherapy						
NCT05468138	Not yet recruiting	II	MSI-H gastric cancer	Sintilimab or nivolumab	Parallel assignment: adjuvant chemotherapy (SOX or XELOX) vs. adjuvant treatment with PD-1 antibody (sintilimab or nivolumab) vs. observation	PD-1 Antibody Adjuvant Therapy for GC Patients With MSI-H After D2 Radical Surgery
NCT04152889	Unknown	II	MSI-H gastric cancer	Camrelizumab	Single group assignment: Camrelizumab in combination with docetaxel+S-1 sequenced by camrelizumab+S-1	A Study to Evaluate Camrelizumab in Combination With Docetaxel +S-1 as Adjuvant Treatment Therapy in Stage III Gastric Cancer
Clinical trials on immune checkpoint inhibitors for MSI-H/dMMR resectable GC in neoadjuvant chemotherapy						
NCT04556253	Not yet recruiting	II	MSI-H/dMMR gastric carcinoma	AK104 (a PD-1/CTLA-4 bispecific antibody)	Single group assignment: AK104	AK104 in Locally Advanced MSI-H/dMMR Gastric Carcinoma and Colorectal Cancer
NCT03257163	Recruiting	II	dMMR gastric cancer	Capecitabine embrolizumab	Single group assignment: Neoadjuvant pembrolizumab plus surgery plus adjuvant pembrolizumab+capecitabine+ radiation therapy	Pembrolizumab, Capecitabine, and Radiation Therapy in Treating Patients With Mismatch-Repair Deficient and Epstein-Barr Virus Positive Gastric Cancer
NCT04006262	Recruiting	II	MSI-H/dMMR esogastric adenocarcinoma	Ipilimumab Nivolumab	Single group assignment: Neoadjuvant ipilimumab+nivolumab plus surgery plus adjuvant nivolumab therapy	Perioperative Association of Immunotherapy (Preoperative Association of Nivolumab and Ipilimumab, Post-operative Nivolumab Alone) in Localized Microsatellite Instability (MSI) and/or Deficient Mismatch Repair (dMMR) Oeso-gastric Adenocarcinoma (NEONIPIGA)
NCT04744649	Recruiting	II	MSI-H gastric cancer	JS001 (recombinant humanized anti-PD-1 monoclonal antibody)	Parallel assignment: XELOX/SOX vs. JS001+XELOX/SOX	Neoadjuvant Immunotherapy and Chemotherapy for Locally Advanced Esophagogastric Junction and Gastric Cancer Trial (NICE)
NCT04795661	Recruiting	II	MSI/dMMR gastric cancer	Pembrolizumab	Parallel assignment: Cohort colorectal cancer (CRC) vs. esogastric cancer vs. endometrial cancer vs. other cancer	Immunotherapy in MSI/dMMR Tumors in Perioperative Setting (IMHOTEP)
NCT04817826	Recruiting	II	MSI-H gastric cancer	Durvalumab Tremelimumab	Single group assignment: T300/D as neoadjuvant (cohort 1) or definitive (cohort 2) treatment for MSI, mismatch repair deficient (dMMR) and EBV-negative resectable GAC/GEJAC	TremelImumab aNd Durvalumab For the Non-operatIve Management (NOM) of MSI-high Resectable GC/GEJC. (INFINITY)

*MSI-H* microsatellite instability-high, *dMMR* deficient mismatch repair, *SOX* capecitabine plus tegafur, *XELOX* capecitabine plus oxaliplatin, *T300/D* tremelimumab 300 mg single administration (day 1) and durvalumab 1500 mg Q4W, *GAC/GEJAC* gastric/gastroesophageal junction cancer

Besides research on immunoadjuvant therapy, immunotherapy is now used for neoadjuvant therapy, representing a good choice for patients with resectable MSI-H/dMMR gastric cancer. On the basis of the rationale of neoadjuvant treatment, more benefits could be obtained by immunotherapy. Indeed, patients are usually in a better physical condition before surgery, which can better mobilize immune function, and more antigens are released during an immune attack due to the presence of the primary tumor, leading to increased sensitivity to immunotherapy. A case series was published in 2020,^57^ including six patients who received neoadjuvant ICIs and surgery for advanced, resectable, and MSI-H gastrointestinal tumors. After radical surgery, pathological responses were observed in all MSI-H/dMMR tumors, with a complete response observed in 83% (*n* = 5/6) of patients, substantiating the efficacy of neoadjuvant immunotherapy in patients with MSI-H/dMMR gastrointestinal tumors. The American Society of Clinical Oncology (ASCO) annual meeting in 2022 reported the final data of the NICHE study,^58^ the first neoadjuvant immunotherapy study in colon cancer. It was found that 100% of dMMR patients responded to the combination of neoadjuvant nivolumab plus ipilimumab, and 97% of dMMR patients achieved a major pathologic response (MPR). Substantial benefits were observed with the double-blocking effect of PD-1 and CTLA4. According to the NCCN guidelines published in 2022,^16^ nivolumab ± ipilimumab or pembrolizumab (preferred) is recommended as a neoadjuvant treatment option for resectable MSI-H/dMMR metastatic colorectal cancer (mCRC). During the GERCOR NEONIPIGA phase II study (NCT04006262),^59^ nivolumab- and ipilimumab-based neoadjuvant therapy was also indicated for MSI-H/dMMR gastric/gastroesophageal junction cancer (G/GEJ) adenocarcinoma, with a pathologic complete response (pCR) of 59%. The feasibility of double-blocking neoadjuvant treatment was demonstrated and associated with a high pCR rate in gastric cancer, suggesting that some patients with MSI-H/dMMR GC may be protected from surgery. The INFINITY study,^60^ an ongoing phase II, multicenter, single-arm, multicohort trial from Italy, was designed to assess the activity and safety of the combination of anti-CTLA4 tremelimumab and the anti-PD-L1 durvalumab as a neoadjuvant treatment for patients with resectable MSI-H/dMMR G/GEJ cancer. Approximately 310 patients underwent the molecular prescreening test. Ultimately, 31 patients were enrolled and classified in cohorts 1 (*n* = 18) and 2 (*n* = 13). Patients in cohort 1 received a 12 week treatment with a single high dose of tremelimumab 300 mg and durvalumab 1500 mg for 4 weeks (T300/D) for three cycles followed by surgery, and cohort 2 investigated non-operative management after the same treatment regimen. The primary endpoint of cohort 1 was pCR rate (ypT0N0) with negative circulating-tumor DNA (ctDNA) status after T300/D neoadjuvant immunotherapy in the resectable MSI-H/dMMR G/GEJ cancer population. The preliminary results have been published in ASCO 2023,^61^ showing that among 15 evaluable patients, the pCR rate was 60% (9/15) and the major-complete pathological response (< 10% viable cells) was 80%. Overall, preoperative T300/D was safe and provided promising proof of biological clinical evidence on the neoadjuvant immunotherapy schedule with ICIs of patients with resectable MSI-H/dMMR G/GEJ cancer. As previously mentioned, neoadjuvant immunotherapy for patients with resectable MSI-H/dMMR GC has also been actively implemented in clinical practice. A case report by Chubenko et al.^62^ illustrated the favorable effects of neoadjuvant chemotherapy on a patient with MSI-H/dMMR GC. Partial remission was observed by computed tomography (CT) examination after four cycles of neoadjuvant therapy with nivolumab. After six cycles of neoadjuvant nivolumab treatment, endoscopic surgery was performed, and postoperative pathology showed no residual tumor cells, achieving pCR. In addition, four prospective clinical trials are currently underway with pembrolizumab used in two experimental groups (Table 5). In the experimental group of the two remaining trials, AK104 (a PD-1/CTLA-4 bispecific antibody) and JS001 are being assessed, respectively. JS001 is a Chinese anti-PD-1 monoclonal injection antibody approved for melanoma. It is highly conceivable that the preoperative treatment paradigm will evolve in the near future as results from these clinical trials evaluating the inclusion of immunodrugs in neoadjuvant therapy become available.

## Discussion

To our knowledge, this is the first review to investigate the heterogeneity and adjuvant therapy regimes in resectable GC with MSI-H/dMMR. We explored the heterogeneity of MSI-H/dMMR with emphasis placed on the regional and pathological stage and conducted an in-depth investigation of the effect of adjuvant chemotherapy and immunotherapy in resectable MSI-H/dMMR GC.

Interestingly, we found significant geographic disparities in MSI-H/dMMR prevalence. In this respect, the prevalence of MSI-H/dMMR was higher in the West than in Asia, suggesting the presence of ethnic differences. This heterogeneity has been validated in early resectable and late GC, attributed to the influence of factors,^63–65^ and has significant clinical implications. From the genetics perspective, it has been reported that genetic polymorphisms of some metabolic enzymes and genes such as cytochrome p450 2E1 (CYP2E1),^63^ glutathione S-transferase mu 1 (GSTM1),^64^ and glutathione S-transferase theta 1 (GSTT1)^65^ are closely related to the development of gastric cancer. As for genes that influence the expression of microsatellites, the significance of the hMLH1 gene promoter has been established. Hypermethylation of the promoter of the hMLH1 gene reportedly plays an important role in mismatch repair during DNA replication and is significantly associated with microsatellite instability.^66^ A total of 71.4% of MSI-positive GC tumors showed hypermethylation, whereas only 29.8% of MSI-negative tumors were hypermethylated at the hMLH1 promoter region. In addition, cigarette smoking and alcohol consumption have been associated with the hypermethylation of the hMLH1 gene promoter, which may increase the possibility of microsatellite instability. A population-based case-control study conducted in Italy involving 126 patients with gastric cancer was carried out by Palli et al. to evaluate the role of dietary risk factors in GC according to MSI statuses.^67,68^ A specific diet pattern was associated with MSI+ gastric cancer, indicating that frequent consumption of fresh fruits and vegetables can greatly reduce the risk of MSI cancer, while high consumption of red meat, meat paste, total protein, and nitrite increased the risk of MSI GCs. A Western-style diet rich in red meat, processed meat (steak, sausage, etc.), and refined compounds (hamburger, bread, etc.) represents a potential cause of the elevated fraction of MSI-H/dMMR among European and American countries. In addition, the high proportion might be attributed to increased alcohol consumption in Western countries. In this respect, a pivotal study showed that the average alcohol consumption of American and Chinese men was 15 L and 10 L, respectively.^69^ The above studies overlap in their assertion that this heterogeneity is influenced by a combination of genetics, dietary habits, and other factors, emphasizing the need for further studies.

Interestingly, we also found that resectable gastric cancers have a higher frequency of MSI-H/dMMR than advanced-stage GC, especially stage II disease, similar to findings reported in colon cancer. Data from the PETACC-3 trial^47^ demonstrated that MSI-H/dMMR is more common in stage II colon cancer than in stage III disease (22% vs. 12%; *P* < 0.0001), and stage IV tumors with MSI-H/dMMR account for the lowest proportion (only 3.5%).^48^

Given that the proportion of MSI-H/dMMR is generally higher in resectable GC than in advanced-stage GC, it is necessary to evaluate the MSI status of each patient. Nonetheless, it remains unclear whether adjuvant chemotherapy is the optimal therapeutic strategy for patients with resectable GC with MSI-H/dMMR after surgery. Regarding the role of MSI-H/dMMR in predicting chemotherapy efficacy in resectable GC, a growing literature suggests a lack of benefit of 5-FU chemotherapy in patients with MSI-H/dMMR, although inconsistent findings have been reported. The mechanisms involved have not yet been elucidated. One significant hypothesis relates to a high tumor mutation burden (TMB-H), which suggests that the increased presence of T cells in MSI-H/dMMR tumors can be stimulated to fight against cancer, leading to a favorable prognosis. Following chemotherapy, immunosuppressive therapy is commonly administered, which contributes to a reduction in tumor-infiltrating lymphocytes (TILs) and consequently promotes resistance to chemotherapy.^70,71^ Furthermore, an in vitro study by Tsai and colleagues suggested that the activation of autophagy could induce MSI-H/dMMR gastric cancer resistance against 5-FU.^36^ These hypotheses for resistance mechanisms to the chemotherapeutic agents have been validated in the effects of adjuvant chemotherapy on MSI-H/dMMR colon cancer. Sargent et al. carried out a retrospective study and illustrated that treatment was associated with reduced overall survival in patients with stage II disease and with MSI-H/dMMR tumors.^72^ Another retrospective study involving patients with stage II and III with MSI-H/dMMR tumor status indicated that patients with tumors exhibiting microsatellite stable (MSS) or MSI-L tended to benefit from fluorouracil-based adjuvant chemotherapy [HR 0.72 (0.53–0.99)], while worse outcomes were observed among patients with MSI-H [HR 2.14 (0.83–5.49)].^73^ Taken together, the above findings suggest that chemotherapy does not represent the optimal approach to adjuvant therapy. Furthermore, tumors with MSI-H/dMMR are susceptible to immunotherapy because of infiltrative immune cells and high tumor burdens. The clinical guidelines published by the Chinese Society of Clinical Oncology^17^ for diagnosing and treating gastric cancer recommend that adjuvant chemotherapy should not be encouraged after radical surgery in MSI-H patients. Instead, these patients should be observed or undergo immunotherapy in clinical trials. Prospective clinical trials such as KEYNOTE059,^74^ KEYNOTE061,^75^ and KEYNOTE062^76^ demonstrated an effective clinical response in patients with advanced MSI-H/dMMR gastric cancer. Accordingly, the FDA approved the application of pembrolizumab and dotalizumab. On the basis of these results, the addition of PD-1 inhibitors to adjuvant therapy may be superior to chemotherapy alone for patients with resectable MSI-H/dMMR GC after radical surgery.

In addition, for patients with stage II GC with the highest prevalence of MSI-H/dMMR, whether neoadjuvant is needed depends on the experience of clinicians, with no theoretical basis currently available. More individualized treatment strategies could be provided to clinicians by exploring the predictive effect of MSI-H/dMMR on the efficacy of neoadjuvant therapy on resectable gastric cancer. In the era of conventional chemotherapy, a relatively large cohort study from New York was performed in 2022^46^ to examine the association between MSI-H/dMMR and survival in patients with resectable gastric cancer receiving chemotherapy, including neoadjuvant, adjuvant, and in combination with radiotherapy and compared with patients treated with surgery alone. The 3 year OS and DFS rates were higher among patients treated with surgery alone, though a survival difference was not observed between patients with neoadjuvant or perioperative chemotherapy and those without (88% vs. 79%, *P* = 0.48 and 78% vs. 73%, *P* = 0.66). It was found that MSI-H/dMMR is associated with a positive prognosis in patients treated with surgery alone and a negative prognosis in patients treated with chemotherapy. These findings suggested that chemotherapy alone exhibited limited efficacy in enhancing surgery’s overall efficacy and improving survival outcomes. With significant inroads achieved in immunotherapy, the development of immune preparations has been accompanied by constant attempts to apply them in neoadjuvant regimes, followed by documentation of cases of gastrointestinal cancer to demonstrate their feasibility. Moreover, preliminary evidence of the predictive value of MSI-H/dMMR on neoadjuvant immunotherapy has been obtained from clinical trials, corroborating that neoadjuvant chemotherapy involving PD-1 inhibitors yields better advantages. With the advent of neoadjuvant immunotherapy, it becomes possible to broaden the scope of applicable neoadjuvant treatments, thereby offering a survival benefit to a greater number of patients.

## Conclusions

Although the past decade has witnessed unprecedented medical progress, gastric cancer remains an important public health issue. The significant increase in studies published on molecular typing has paved the way to address specific therapeutic strategies. The systematic classifications of GC, such as TCGA and ACRG, have substantiated the important value of the MSI genotype. Significant heterogeneity in the frequency of MSI-H/dMMR among resectable GCs has been found in our literature review, mainly attributed to the heterogeneity in geographic distribution and pathological stages. In the meantime, although the limited studies in the adjuvant treatment of MSI-H/dMMR gastric cancers have been deemed inadequate to determine an explicit treatment regimen after surgery, most studies substantiate the low chemosensitivity of MSI-H/dMMR patients. Additionally, as the treatment schemes of immunotherapy in advanced GCs have been guideline endorsed, their value in adjuvant and neoadjuvant settings has been emphasized. Despite the small number of patients with MSI GC enrolled in the available RCTs and the lacking of prospective studies, our review aimed to point out that better responses might occur if immunotherapy is offered earlier for patients with MSI-H/dMMR gastric cancer. This promising area requires further study, which will hopefully shed more light on the optimal clinical regimen to improve the outcomes of this patient population.

### Supplementary Information

Below is the link to the electronic supplementary material.

Supplementary file1 (DOCX 222 KB)
