# Supplementary material for: Heterogeneity and Adjuvant Therapeutic Approaches in MSI-H/dMMR Resectable Gastric Cancer: Emerging Trends in Immunotherapy
Source: Ann Surg Oncol. 2023 Sep 4;30(13):8572–87. doi: 10.1245/s10434-023-14103-0 (PMC10625937; doi:10.1245/s10434-023-14103-0)
Supplement: Supplementary file 1 — Supplementary file1 (DOCX 222 KB) [file 10434_2023_14103_MOESM1_ESM.docx]

**Supplementary Materials**

**Supplementary Methods**

**Literature Sources and Search Strategy**

A literature research was performed in PubMed and Web of Science from January 1996 to June 2022, according to the Preferred Reporting Items for Systematic Reviews and Meta-Analysis (PRISMA) guidelines. The search terms were as follows:("Replication Error Phenotype"[tw] OR "Microsatellite Instability"[Mesh] OR "DNA Mismatch Repair"[Mesh]) AND ("Stomach Neoplasms"[Mesh] OR "Gastric Neoplasm"[tw] OR "Gastric Cancer"[tw]). The consort diagram is shown in Figure S1.

**Data Extraction**

The detailed information was extracted from the included studies if available: study region, tumor stage and the number of dMMR/MSI-H gastric cancer cases for exploring the heterogeneity of geographical distribution and pathological stages. Besides, the study also extracted Disease Free Survival (DFS), Overall Survival (OS) and hazard ratios (HRs) with 95% confidence intervals (CIs) of adjuvant chemotherapy versus surgery alone for the dMMR/MSI-H patients, and Overall Response Rate (ORR) or Progression Free Survival (PFS) of immunotherapy for dMMR/MSI-H patients versus the MSS/MSI-L patients.

**Statistical Analysis**

Two different statistical approaches were used in our review. (1)The comparisons of dMMR/MSI-H proportion between Asia and Europe were analyzed using the unpaired t-test after a normal distribution by Shapiro-Wilk test (S-W test). (2) The analysis of differences between Northern and Southern China vs. Korea vs. Japan was performed using one-way analysis of variance (one-way ANOVA). A two-sided P value lower than 0.05 was considered statistically significant. All analyses were performed using SPSS version 26.0 for Windows (SPSS Inc., Chicago, IL, USA) and GraphPad Prism (version 9.0).

**Figure S1**


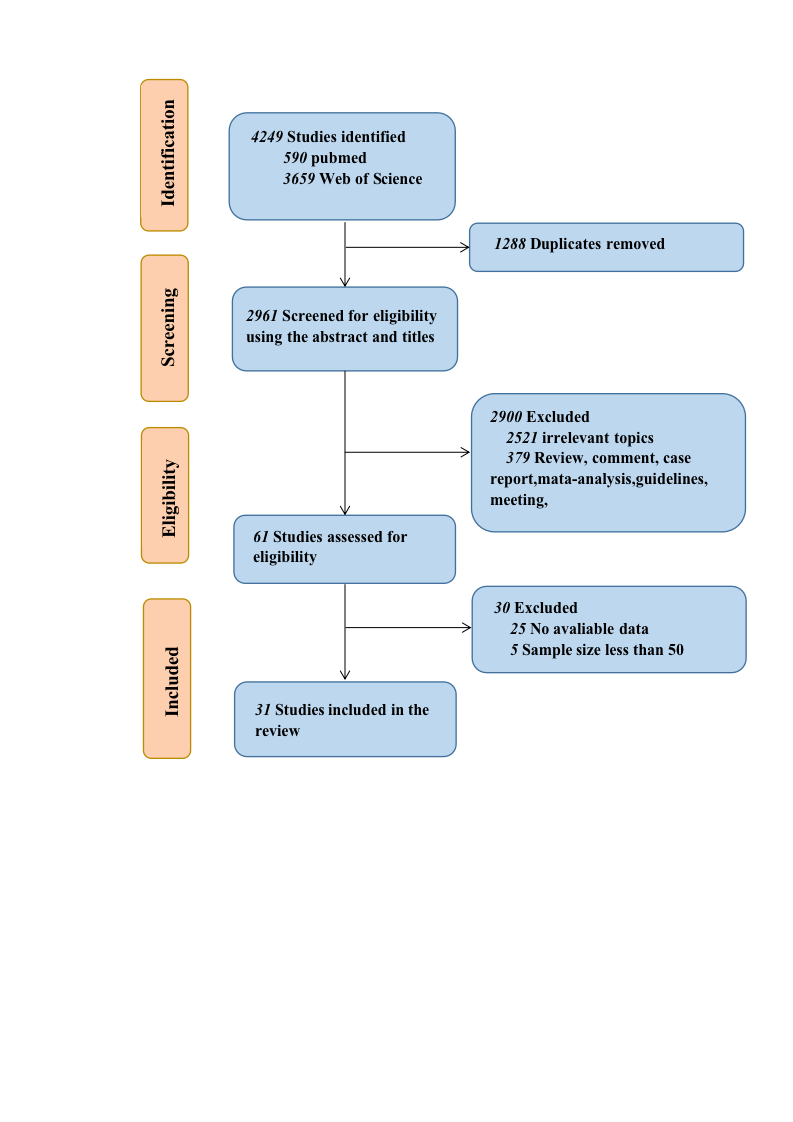


**Figure S1** Study flow diagram. Thirty-one studies were identified for analyzing the heterogeneity of geographical distribution and pathological stages.

**Figure S2**


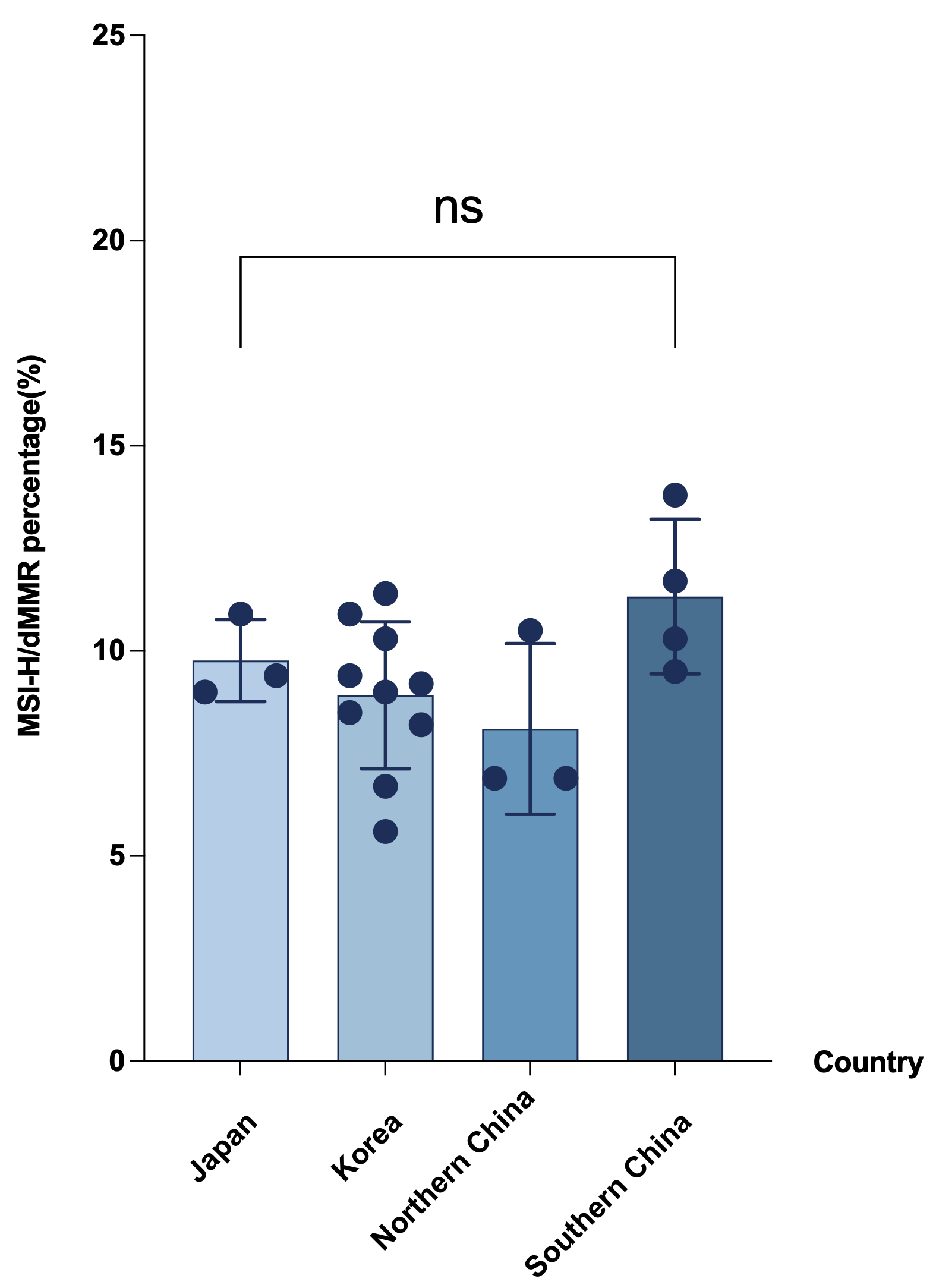


**Figure S2** The differences of MSI-H/dMMR proportion within Asian states(Japan vs. Korea vs. Northern China vs. Southern China) (P=0.106).MSI-H:Microsatellite Istability-High; dMMR: deficient Mismatch Repair
